# Bone marrow-derived mesenchymal stromal cells in necrotizing enterocolitis treatment: a narrative review

**DOI:** 10.3389/fped.2025.1624236

**Published:** 2025-07-31

**Authors:** Andrea Tomaselli, Matteo Tripodi, Livia Provitera, Genny Raffaeli, Stefania Crippa, Ludovica Raymo, Carolina Vittoria Bronzoni, Ludovica Santi, Cristina Arribas, Monica Fumagalli, Stavros Polydoros Loukogeorgakis, Maria Ester Bernardo, Felipe Garrido, Giacomo Cavallaro

**Affiliations:** ^1^ Neonatal Intensive Care Unit, Fondazione IRCCS Ca' Granda Ospedale Maggiore Policlinico, Milan, Italy; ^2^ Department of Clinical Sciences and Community Health, Università degli Studi di Milano, Milan, Italy; ^3^ San Raffaele Telethon Institute for Gene Therapy (SR-Tiget), IRCCS San Raffaele Scientific Institute, Milan, Italy; ^4^ Department of Pediatrics, Clínica Universidad de Navarra, Madrid, Spain; ^5^ Great Ormond Street Institute of Child Health, University College London, London, United Kingdom; ^6^ Neonatal and Paediatric Surgery Department, Great Ormond Street Hospital for Children NHS Foundation Trust, London, United Kingdom; ^7^ Pediatric Immunohematology Unit and BMT Program, IRCCS San Raffaele Scientific Institute, Milan, Italy; ^8^ Maternal and Child Department, Vita-Salute San Raffaele University, Milan, Italy

**Keywords:** necrotizing enterocolitis, bone marrow-derived mesenchymal stromal cells, toll-like receptor 4, exosomes, secretome

## Abstract

Necrotizing enterocolitis (NEC) presents a life-threatening intestinal emergency primarily affecting premature infants in neonatal intensive care units. This disease is a significant cause of morbidity and mortality in such newborns. NEC involves inflammation, bacterial overgrowth, and cell death affecting a portion of the bowel wall, commonly the distal ileum. Despite advances in neonatal care, the pathogenesis of NEC remains not fully understood. Although its pathogenesis remains not fully elucidated, the upregulation of Toll-like receptor 4 in the premature intestinal epithelium is recognized as a key factor contributing to epithelial barrier dysfunction. Recent studies have explored the potential of mesenchymal stromal cells (MSCs) in NEC management. MSCs are up-and-coming candidates for preclinical NEC models as they possess anti-inflammatory and immune modulatory properties, which reduce inflammation, help increase intestinal integrity, and help tissue repair. Bone marrow-derived mesenchymal stromal cells (BM-MSCs) have proven impactful in most experimental settings, mitigating injury from NEC and facilitating intestinal development. While MSC therapies hold promise, challenges remain regarding inconsistent isolation and expansion of these cells, variable differentiation, and possible tumorigenicity *in vivo*. As a result, the focus has been drawn to MSC-derived secretome, especially exosomes, as a novel cell-free therapeutic. These bioactive molecules transported by exosomes can reduce inflammation and facilitate tissue repair, providing a safer and more plausible alternative to treating NEC. Further research is needed to standardize secretome production and evaluate its clinical efficacy and safety. This review aims to provide a comprehensive overview of the mechanisms of action and the available research on human (h)BM-MSCs to support the development of studies that may prevent and/or treat the disease.

## Highlight

1. Numerous preclinical studies in murine models have demonstrated the efficacy of bone marrow-derived mesenchymal stromal cells (BM-MSCs) in preventing and treating necrotizing enterocolitis (NEC). However, their use inevitably raises ethical, legal, and biological concerns that require careful consideration.
2. This review provides an overview of exosomes and secretome as viable alternatives that effectively address the aforementioned limitations.
3. This review endeavors to furnish an up-to-date and comprehensive synopsis of the current body of literature, aiming to equip clinicians and researchers with a thorough understanding while also considering the potential for imminent clinical translation.

## 1 Introduction

Necrotizing enterocolitis (NEC) is a severe inflammatory condition that primarily affects the terminal ileum and is most commonly observed in preterm infants (1). The severity of NEC ranges from mild mucosal injury to transmural necrosis with bowel perforation, and it is associated with high morbidity and mortality rates in neonatal intensive care units (NICUs) (2–4).

The pathogenesis of NEC is multifactorial, involving the underdeveloped intestinal barrier, dysregulated immune responses, and microbial dysbiosis. Notably, upregulation of Toll-like receptor 4 (TLR4) in the premature gut plays a pivotal role in NEC development by impairing epithelial barrier integrity and promoting inflammation (5–9). Aberrant inflammatory signaling and the subsequent release of cytokines further compromise the intestinal lining, making the tissues more susceptible to ischemic injury. Additional contributing factors, such as impaired blood flow and compromised oxygen delivery to the immature intestinal epithelium, exacerbate tissue damage and may culminate in extensive tissue death if left untreated (10, 11).

Despite advancements in neonatal intensive care management, NEC often results in short- and long-term complications, with a profound impact on the affected infant's life (12, 13).

Emerging therapies, specifically mesenchymal stromal cells (MSCs) therapy, have demonstrated potential for prevention and treatment options for NEC. MSCs are multipotent cells capable of differentiating into multiple lineages and secreting a broad range of paracrine factors that support tissue repair (14). In particular, bone marrow-derived MSCs (BM-MSCs) have shown potential in preclinical NEC models, significantly reducing intestinal injury and inflammation. The therapeutic effects of MSCs are primarily mediated through their secretion of anti-inflammatory cytokines and their ability to modulate the endogenous repair process by inducing epithelial regeneration and barrier function (15).

However, several challenges must be addressed before MSC-based therapies can be successfully translated into clinical practice for NEC. These obstacles include variability in MSCs isolation and expansion, limited engraftment efficiency, and potential risks such as tumorigenicity and immune rejection (16–18). Furthermore, the *in vivo* behavior of MSCs remains incompletely understood, and concerns persist regarding their long-term safety and efficacy (19).

In this context, there is growing interest in cell-free therapeutic approaches, particularly focusing on the MSC-derived secretome, including exosomes and other paracrine factors (20, 21). These strategies offer several advantages, such as a reduced risk of tumorigenicity, simplified manufacturing and storage process, and the potential to harness the therapeutic benefits of MSCs without the complexities associated with cellular therapies.

Despite advances in supportive care, NEC remains a life-threatening condition in preterm infants, associated with significant morbidity and mortality. Innovative therapies, including MSC-based and secretome-based interventions, may provide more effective and targeted strategies for preventing and treating NEC (22–33).

This review aims to summarize the current knowledge regarding BM-MSCs' mechanisms of action, highlight their biological effects in the context of NEC, and discuss the challenges and opportunities for their preclinical and clinical translation, thereby fostering the development of novel therapeutic strategies.

## 2 NEC overview

### 2.1 Epidemiology

The incidence of NEC varies from 0.3 to 2.4 per 1,000 live births, with geographic, ethnic, and neonatal care strategy variations (34–36). The incidence of the disease is strongly inversely correlated with the gestational age (GA) at birth, with approximately 90%–95% of cases occurring in infants born before 36 weeks' GA (1). A multicenter cohort analysis conducted in the United States revealed only marginal improvement in the overall incidence of NEC cases among Very Low Birth Weight (VLBW) over the past years, with a decrease from 9% in 2006 to 6% in 2017 (37). However, term infants can also be affected, mainly when associated with other conditions such as congenital heart disease, polycythemia, early-onset bacterial sepsis, hypotension, or maternal drug use (38, 39).

### 2.2 Risk factors and pathogenesis

Since its description in the 1940s to 1950s, NEC is recognized as a multifactorial disease caused by various risk factors, including prematurity, formula feeding, microbial dysbiosis, immature intestinal barrier, underdeveloped motility, immune regulation, hypoxia, inadequate microcirculation, and intense inflammation (Table 1) (40–57). In this context, the upregulation of TLR4 is pivotal in NEC pathogenesis, directly contributing to the intestinal epithelial barrier impairment (5–7). Because of the central role of TLR-4 in the pathogenesis of the disease, many therapeutic options are moving toward drugs explicitly targeting the signaling pathway associated with this molecule. The inhibitor 2-acetamidopyranoside (C34), identified by Neal et al., mainly exhibits efficacy in inhibiting TLR-4 signaling both *in vitro* and *in vivo*. Mice treated with C34 showed preserved intestinal mucosa and a reduced incidence of the disease in an experimental model of NEC (58, 59). The pregnane X receptor (PXR), a xenobiotic sensor that acts as an intermediary for specific host-bacterial metabolites, can inhibit the TLR-4 pathway (60). In PXR knockout mice, symptoms following NEC induction were more severe. Activation of intestinal PXR by lithocholic acid (LCA), a PXR agonist, reduces NEC-induced intestinal inflammation (60, 61). Furthermore, Dai et al. recently demonstrated that high mobility group box 1 (HMGB1), a DNA-binding protein, inhibits enterocyte migration by activating the TLR-4 pathway. Hence, NEC is associated with elevated protein expression (62). In a rat model, glycyrrhizin, an HMGB1 inhibitor, was used to reduce the disease incidence through the TLR-4 and nuclear factor kB (NF-kB) pathways (63).

**Table 1:** Principal risk factors involved in NEC development.

**Prematurity.**
**Formula feeding.**
**Bacterial infection.**
− Patterns of colonization or uncontrolled growth. − Microorganism pathogenicity
**Imbalance in inflammatory response.**
− Enhanced pro-inflammatory response. − Suppression of anti-inflammatory sources
**Intestinal barrier compromission.**
− Physical barrier (skin, mucous membranes, epithelium, microvilli, tight junctions, mucus) − Immunity factors (neutrophils, macrophages, eosinophils, lymphocytes, secretory IgA). − Biochemical factors [antimicrobial proteins (defensins, cryptids), oligosaccharides, glutamine, lactoferrin, polyunsaturated fatty acids, nucleotides, growth factors (EGF, TGF, IGF, EPO), gastric acids, cytokines]
**Reduced intestinal motility.**
− Migrating Motor Complex (MMC)
**Alterations in autoregulation of intestinal circulation.**
EGF: epidermal growth factor; EPO: erythropoietin; IgA: Immunoglobulin A; IGF: insulin growth factor; TGF: transforming growth factor.

The disruption of the TLR4 pathway and the other risk factors are closely linked to alterations in the normal proliferation/apoptosis cycle within the intestinal tissue. Specifically, the Wingless and Int-1 (Wnt) signaling pathway, originating from Paneth cells, drives crypt division and plays a pivotal role (64–66). After apoptosis, which leads to the average turnover of epithelial cells, there is an autophagic clearance of apoptotic remnants and shedding of cell remnants into the lumen (66). Under normal conditions, there is a balance between cell proliferation and loss through processes such as apoptosis, autophagy, and shedding. In contrast, in experimental models and humans, NEC is defined by intestinal necrosis, epithelial cell loss, and increased apoptosis and autophagy (67–70). The loss of this delicate intestinal stem cell niche disrupts the balance between cell propagation and loss, while also impairing cell migration (71). Intestinal epithelial apoptosis triggers severe bowel necrosis in an experimental neonatal rodent model that resembles necrotizing enterocolitis in human newborns (72).

### 2.3 Diagnosis and treatment

Clinical features, radiographic findings, and laboratory values based on Bell's staging criteria support the diagnosis of NEC. They were initially described in 1978 and subsequently refined by Walsh and Kliegman in 1986, extending the original 3-stage classification to the current 6-stage classification (73–75). Despite their well-recognized pitfalls, Modified Bell's criteria are widely employed to stage the severity of NEC, with higher stages associated with a greater risk of adverse outcomes (74).

The condition's severity is commonly classified into “medical NEC” vs. “surgical NEC”, a distinction that has critical implications, as surgically managed disease carries higher mortality and worse long-term outcomes (76).

In clinical practice, the early manifestation of NEC typically shows wide variation in infants, and its non-specific nature makes it challenging to make an early diagnosis. The disease exhibits various clinical symptoms and signs, including apnea, bradycardia, lethargy, irritability, and temperature instability. This holds for other conditions in preterm infants, such as sepsis (59, 77). Concurrent gastrointestinal symptoms, such as abdominal distension, blood in the stools (either gross or occult), gastric residual volumes, and bilious vomiting, may also support the diagnosis of NEC (78, 79). Radiographic findings and laboratory values help evaluate diseases and exclude other conditions from the differential diagnosis of NEC (80). Abdominal x-rays can indicate intestinal dilation, ileus, pneumatosis intestinalis, portal venous gas, or pneumoperitoneum, which is almost pathognomonic for NEC (81, 82). Additionally, abdominal ultrasound is increasingly recognized as a promising method for enhancing NEC diagnosis and guiding clinical decision-making (83, 84).

Acute complications include fulminant sepsis, peritonitis, abscess formation, thrombocytopenia, granulocytopenia, disseminated intravascular coagulation (DIC), and hypotensive shock (85, 86).

NEC medical management involves implementing measures such as bowel rest, nasogastric or orogastric drainage, fluid balance, parenteral nutrition, pain medication, and the empiric administration of broad-spectrum antibiotics. Critically affected patients often require ventilatory support, vasopressors for blood pressure stabilization, and blood transfusions (87, 88).

While numerous NEC cases are treated medically, around 20%–70% of affected infants require surgical procedures (89).

The only definitive indication for surgical intervention is the presence of gastrointestinal tract (GIT) perforation, as determined by radiographic evidence of pneumoperitoneum or paracentesis showing enteric content (90). However, surgery may be required when infants do not display improvement despite medical management or in patients with particular symptoms (91, 92).

Surgical management includes placing a peritoneal drain or performing a laparotomy, resecting non-viable segments of the gastrointestinal tract, and potentially creating ostomies or primary anastomoses (93–97).

### 2.4 Short- and long-term outcomes

Despite significant advances in neonatal intensive care, NEC-related mortality rates remain alarmingly high, peaking at up to 42% in infants weighing less than 750 g at birth (98, 99). Patients with “medical NEC” have a 21% mortality rate, while those with “surgical NEC” face a mortality rate of up to 35%–50% (100, 101). Additionally, infants with extensive gut resection often face complications such as wound dehiscence, nutrient imbalance, a high rate of abscesses, a high incidence of strictures, and short bowel syndrome (12, 76, 100, 102). Moreover, extended use of total parenteral nutrition (TPN) raises the risk of infections, cholestasis, and liver failure (98, 103). Additionally, NEC patients have been shown to experience worsened neurological outcomes in both the short and long term (13, 104).

### 2.5 Preventive strategies

NEC research should emphasize prevention over treatment, which can alter outcomes and reduce morbidity. Preventive strategies have centered on standardized protocols, breastfeeding and nutrition with human milk, antenatal maternal steroid administration, prophylactic probiotics, careful antibiotic use, and avoiding histamine type II receptor antagonists (22–29, 31–33, 105).

## 3 Stem and stromal cells in NEC prevention and therapy

Stem and stromal cells are unspecialized cells that can self-renew and differentiate into various cell types (106). They can be categorized as totipotent, pluripotent, and unipotent based on their differentiation capabilities (107). Totipotent cells can differentiate in various directions; pluripotent cells can develop into different tissues, while unipotent cells can only change into one cell type (108). The intestinal epithelium renews rapidly, typically every 4–5 days. Stem cells located in the crypts divide swiftly and differentiate into various cell types, including enterocytes, enteroendocrine cells, goblet cells, and Paneth cells, before migrating to the tips of the villi (109).

Stem cells possess anti-inflammatory properties and can improve tissue healing in various disease models (110). Similarly, preclinical studies have observed encouraging outcomes regarding their application in the therapy and prevention of NEC (111). Table 2 summarizes the type of stem cells, their origin (including the route of administration), cell concentration, timing of administration, animal model (including age), and protocol duration. Substantial heterogeneity in methodologies and experimental designs is evident across studies.

**Table 2:** Different types of stem cells used for NEC treatment.

Type of stem cells	Stem cells origin (route of administration)	Cell concentration (day of administration)	Animal model (age)	Duration of protocol	Reference
Amniotic fluid-derived stem cells	Rat (i.p. injection)	2 × 10^6^ (P6-P7)	C57BL/6 Mice (P5)	9 days	Li et al. (112)
	Rat (i.p. injection)	2 × 10^6^ (P3–4, P6–7, depend on groups)	C57BL/6 Mice (P5)	9 days	Li et al. (113)
	Rat (i.p. injection)	2 × 10^6^ (P1)	Rat (P0)	4 or 15 days (based on groups)	Zani et al. (68)
	Rat (i.p. injection)	2 × 10^6^ (P0)	Rat (P0)	2 days	McCulloh et al. (114)
Amniotic fluid neural stem cells	Rat (i.p. injection)	2 × 10^6^ (P0)	Rat (P0)	2 days	McCulloh et al. (114)
	Rat (i.p. injection)	2 × 10^6^ (P0)	Rat (P0)	4 days	McCulloh et al. (115)
	Rat (i.p. injection)	2 × 10^6^ (P3–P4)	C57BL/6 Mice	9 days	Li et al. (116)
Bone marrow mesenchymal stem/stromal cells	Rat (i.p. injection)	2 × 10^6^ (P0)	Rat (P0)	2 days	McCulloh et al. (114)
	Human (i.p. injection)	6 × 10^5^ (P3)	Rat (P0)	4 days	Tayman et al. (117)
	Human (i.p. injection)	0,5 × 10^6^ and 1 × 10^6^ (based on groups)	C57BL/6 Mice (P2)	5 days	Provitera et al. (15)
Umbilical cord-derived stem cells	Human (i.p. injection)	80′000 cells/g (P6)	Mice (P5)	9 days	Di et al. (108)
Placental-derived mesenchymal stem cells	Human (i.p. injection)	2 × 10^6^ (P2)	Rat (P0)	4 days	Di et al. (108)
Enteric neural stem cells	Rat (i.p. injection)	2 × 10^6^ (P0)	Rat (P0)	4 days	McCulloh et al. (115)

### 3.1 Mesenchymal stromal cells

MSCs are a versatile type of adult stem cell (ASCs) localized in specialized niches within various tissues throughout the body, including the bone marrow, adipose tissue, and umbilical cord. MSCs play a crucial role in tissue homeostasis, repair, and regeneration. They are multipotent cells capable of differentiating into several cell types, including bone, cartilage, fat, and connective tissue, making them a valuable resource for regenerative medicine (14).

MSCs can be easily isolated *in vitro* by plastic adherence and expanded in culture as fibroblast-like cells expressing CD90, CD73, and CD105 to reach an appropriate number for clinical use (14, 118). Also, they need to lack the expression of hematopoietic cell surface markers CD34, CD45, CD11a, CD19, and HLA-DR and to adhere to plastic surfaces under culture conditions (119, 120). Clinical and preclinical data reported the therapeutic benefits of MSC transplantation in repairing injured tissues and modulating the inflammatory response to damage (121, 122). Importantly, due to the lack of HLA class II and co-stimulatory molecule expression, MSCs are immune-evasive, enabling MSC transplantation across histocompatibility barriers and the creation of off-the-shelf therapies, consisting of exogenous MSCs in culture (123).

One of the defining characteristics of MSCs is their immunomodulatory and anti-inflammatory properties (124). They can sense inflammatory signals via TLRs and secrete multiple anti-inflammatory molecules to support tissue regeneration, regulate immune responses to protect tissue from excessive damage, and promote the survival of tissue-derived precursor cells when injected in response to injury (125–137).

MSCs interact with and regulate the activity of innate and adaptive immune cells, inhibiting the proliferation of B, T, and natural killer (NK) cells through direct interactions and the release of soluble molecules, such as transforming growth factor (TGF)-β1, indoleamine 2,3-dioxygenase (IDO), and prostaglandin E2 (PGE2). Consequently, these molecules halt immune cells in the G0 stage to prevent their proliferation. Additionally, MSCs modulate T-cell activation by suppressing the production and secretion of inflammatory cytokines and preventing dendritic cells' activation and maturation (132, 138).

For this reason, MSCs are frequently used in patients to prevent heightened immune cell activation and to modulate the inflammatory response, promoting tissue regeneration in conditions like Crohn's disease and graft-versus-host disease (GvHD) following allogeneic hematopoietic stem cell transplantation (139, 140). MSCs have also been utilized clinically in regenerative medicine due to their ability to differentiate into bone and chondrocytes, thereby repairing cartilage and similar tissues (141). Recent findings increasingly suggest that the paracrine activity of MSCs is the primary mechanism underlying their therapeutic efficacy. Rather than relying solely on direct differentiation, MSCs promote tissue repair primarily through the secretion of paracrine factors that enhance the survival of injured cells and activate tissue-resident progenitor populations, thereby preventing the formation of non-functional scar tissue (142–145). Their regenerative potential is therefore attributed to modulation of the stem cell niche and secretion of bioactive molecules, including anti-inflammatory cytokines, immunoregulatory mediators, and growth factors such as insulin-like growth factor 1 (IGF-1), vascular endothelial growth factor (VEGF), hepatocyte growth factor (HGF), and basic fibroblast growth factor (bFGF), all of which contribute to processes such as angiogenesis, cell survival, and proliferation (131, 134). A recent review by Che et al. emphasized the invaluable role of MSC-derived paracrine signaling in inflammatory bowel disease (IBD), highlighting how secreted bioactive molecules, such as HGF, VEGF, and epidermal growth factor (EGF), contribute to epithelial repair, angiogenesis, and mucosal regeneration (146). Furthermore, MSCs have been shown to enhance the expression of tight junction proteins (e.g., zonula occludens 1 (ZO-1), occludin), promote goblet cell function and mucosal healing, and stimulate the proliferation and differentiation of intestinal epithelial cells (146). Collectively, these findings suggest that MSCs also represent a promising therapeutic approach for NEC. In support of this hypothesis, several preclinical studies have demonstrated that MSC treatment significantly reduces the severity of NEC in rodent models (114, 115). The beneficial effects observed in these studies are thought to be mediated mainly by MSC-derived paracrine factors, including interleukin (IL)-6, IL-10, and TGF-β, which contribute to the resolution of inflammation and restoration of the intestinal barrier (147–149).

### 3.2 Bone marrow–derived mesenchymal stromal cells

McCulloh et al. have revealed that various types of stem cells exert a therapeutic effect by enhancing the intestinal barrier and decreasing intestinal permeability. However, it remains unclear whether the improved gut barrier function is influenced by common or unique pathways among different stem cell types (114).

BM-MSCs are a subpopulation of MSCs found in the medullary stroma of bone marrow. The characteristics of BM-MSCs are closely linked to the age and condition of the donors, as the quantity and differentiation ability decline with age (150). BM-MSCs derived from young and old donors (under 18 years and over 55 years) exhibit significant differences. The old BM-MSCs show increased expression of β-Gal, a hydrolase found in the lysosomes of aging cells, making it a marker of cellular senescence. Additionally, they display changes in cytoskeleton composition, which correlate with impairments in migration and the ability to respond to biological and mechanical signals (151). Even BM-MSCs from aged mice show a greater presence of cellular senescence markers, including the DNA double-strand break marker phosphorylated H2A histone family member X (γH2AX) and the DNA checkpoint response mediator p53-binding protein 1 (53BP1), compared to those from younger mice, which can enhance osteogenic activities and migration in mice instead (152). BM-MSCs release various paracrine factors that regulate fibrosis, proliferation, apoptosis, and angiogenesis in damaged tissues, thereby promoting the recovery of the injured area, modulating the immune system, and safeguarding cells from apoptosis (153). Their potential in treating neonatal diseases has been the subject of considerable research (154, 155). The administration of MSCs has been shown to be beneficial for inflammatory bowel disease. In animal models of colitis, MSC therapy not only reduced the Th1 cytokine response and enhanced the regulatory T cell (Treg) response. Furthermore, MSC therapy has been demonstrated to improve survival and quickly correct weight loss (156).

Kagia et al. have demonstrated that BM-MSCs can treat colitis in mice and improve overall survival compared to the control group. Additionally, colon health in the treated group was significantly better (157).

In a recent study, Abbuehl and colleagues found that freshly isolated murine BM-MSCs, unlike *ex vivo* expanded ones, can repair stromal niche damage after irradiation and enhance the engraftment of hematopoietic stem and progenitor cells (HSPCs) when co-transplanted intra-bone (158).

The administration of BM-MSCs has effectively reduced the incidence and severity of NEC in a murine model. Specifically, mice with NEC treated with BM-MSCs exhibited decreased levels of Caspase 3, a marker associated with apoptosis, and increased expression of ZO-1. ZO-1 is a protein found at the tight junctions of the intestinal barrier, and its elevated levels compared to the control group suggest that MSCs can help restore the proper functionality of the barrier. These findings indicate that BM-MSCs may have significant therapeutic potential for treating NEC in humans (15).

A study by Seok Lee and colleagues explored oral BM-MSC administration for premature infants to reduce invasive procedures. Administering BM-MSCs orally and intraperitoneally in neonatal mice reduced NEC-related histological injury after NEC induction. The TdT-mediated dUTP Nick-End labeling (TUNEL) assay assessed apoptosis levels, revealing decreased apoptosis in BM-MSC groups. Consequently, stem cell administration lowered TLR-4 expression (159). Previous studies have shown that stem cells can effectively combat NEC in mouse models, regardless of whether BM-MSCs are given before or after NEC onset. These findings suggest that BM-MSCs have potential for preventive and therapeutic uses, achieving notable results whether administered early or later. Additionally, the effects of BM-MSCs are similar whether given orally or intraperitoneally for NEC (159). Wang et al. argued that intraperitoneal injection is the best method for delivering stem cells to treat colitis, despite being an invasive procedure (160). The oral treatment administration is advantageous for vulnerable patients and deserves consideration (159).

Combining BM-MSCs with other factors can enhance their therapeutic potential in NEC prevention by improving their protective efficacy and modulating transcription factors that either suppress or increase the activity of BM-MSCs.

Heparin-binding EGF-like growth factor (HB-EGF) was initially recognized as a possible treatment for NEC almost twenty years ago (161). It safeguards intestinal injury by preventing injury to intestinal stem cells from damage, facilitating enterocyte proliferation and migration, enhancing gut barrier function, and minimizing intestinal apoptosis (162–165). Yang et al. investigated the potential for attaching HB-EGF to BM-MSCs to influence the activity of the stem cells. HB-EGF was found to enhance the proliferation and migration of BM-MSCs while simultaneously reducing their apoptosis. Furthermore, in a rat model of NEC, administering HB-EGF and BM-MSCs decreased the incidence of pathology and lowered levels of 70 kDa FITC-Dextran (FD70), a marker of intestinal permeability, compared to the control and stem cell-only groups (166).

A study by Chen et al. demonstrated that inhibiting the gene responsible for prolyl hydroxylase 2, which plays a role in activating hypoxia-related transcription factors, prompted BM-MSCs to enhance their paracrine effects by releasing protective factors such as TGF-β2. Additionally, the inhibition of prolyl hydroxylase 2 increases survival rates in rats with NEC treated with BM-MSCs by promoting epithelial regeneration (167).

The transplantation of MSCs in animal models of experimental NEC has proven feasible, safe, and effective. Notably, it has been reported that various types of stem cells can restore the integrity of the intestinal barrier (114). MSCs have been shown to protect against intestinal damage and reduce the incidence of NEC (168). Whether stem cell transplantation serves as a preventive or protective model for NEC is yet to be determined.

### 3.3 Alternative stem cell sources for treating NEC

Despite the importance of BM-MSCs as a potential therapy for treating NEC, several other stem cell types show characteristics that may be beneficial in managing intestinal diseases. Stem cells are typically classified based on their origin and differentiation potential.

Embryonic stem cells (ESCs), derived from the inner cell mass of blastocyst-stage embryos, represent the gold standard of pluripotency (169). They can differentiate into all three germ layers (ectoderm, mesoderm, and endoderm), thus giving rise to a broad range of specialized cell types, including neurons, muscle cells, and different tissue-specific cells (170–172). Furthermore, they hold potential for personalized cell-based therapies, where patient-specific pluripotent stem cells can generate replacement tissues or organs (173). However, their clinical application remains ethically controversial, due to their derivation from the human embryo (174). Amniotic fluid-derived stem cells (AF-SCs) represent a promising alternative. These cells exhibit both mesenchymal markers (CD29, CD44, CD90) and embryonic-like characteristics, such as the stage-specific embryonic antigen (SSEA-4) and the octamer-binding transcription factor 4 (Oct4) (175). Their pluripotency, combined with easier collection and culture methods compared to ESCs, makes them particularly attractive for clinical applications. In NEC models, AF-SCs have shown protective effects by enhancing intestinal barrier function through modulation of endoplasmic reticulum stress and upregulation of tight junction proteins like claudin-7 (112). Both enteral administration and intraperitoneal injection of AF-SCs have shown efficacy in experimental NEC models (68, 176).

The enteric nervous system (ENS), often referred to as the “second brain”, interacts with the diverse range of microbes that populate the gastrointestinal tract, forming critical linkages with intestinal microbiota, the immune system, and the endocrine system to maintain a stable intestinal environment (177–179). It undergoes significant damage during the NEC development process, thus increasing the interest in neural stem cells (NSCs) as a potential therapy for NEC.

Neural stem cells, also derived from amniotic fluid (AF-NSCs), express nestin, an intermediate filament protein that can be considered a marker of immature neural cells (180). Despite their slow growth rate when cultured *in vitro*, AF-NSCs have shown potential in treating NEC through Wnt-dependent mechanisms (112, 181, 182).

Neonatal enteric neural stem cells (N-ENSCs) are specialized stem cells found within the ENS that control the complex network of neurons in the gastrointestinal tract. N-ENSCs can differentiate into various neural cell types within the ENS, including neurons and glial cells (183). N-ENSCs have emerged as a promising avenue for research as they can potentially repair or regenerate the ENS's damaged neural and glial cells. This regenerative capacity offers hope for developing innovative treatments for NEC (184).

Beyond BM-MSCs, other MSC sources have also gained attention. Umbilical cord-derived mesenchymal stem cells (UC-MSCs) are multipotent stem cells that can be isolated in a non-invasive manner and exhibit regenerative properties comparable to BM-MSCs, making them a promising alternative for stem cell research and therapies (185). In NEC models, UC-MSCs can promote intestinal integrity by activating endothelial nitric oxide synthase and secreting hydrogen sulfide (108). Placental-derived MSCs (P-MSCs) have also been shown to be a good source of stem cells with potent anti-inflammatory effects (186), able to promote intestinal regeneration via the Wnt/β-catenin pathway (108).

These diverse stem cell populations, ranging from pluripotent ESCs to more specialized AF-SCs and neural stem cells, offer multiple avenues for regenerative medicine approaches, particularly for intestinal pathologies such as NEC. Their varying degrees of potency, combined with distinct mechanisms of action ranging from barrier reinforcement to neural regeneration, could expand the knowledge aimed at the development of novel cell-based therapies. Continuous research into stem cells could lead to a deeper understanding of their therapeutic potential and the resolution of associated ethical issues.

## 4 Mesenchymal stromal cells: the dark side

Preclinical studies involving MSCs in various diseases have generated significant interest and optimism among researchers and patients globally. However, translating these findings into clinical applications presents challenges due to ethical concerns, technical limitations, and potential adverse effects (106, 108).

One of the primary obstacles to the clinical use of adult MSCs is their isolation method. Harvesting MSCs, particularly from the bone marrow, often necessitates invasive procedures, posing a risk to donors and complicating broader clinical implementation (187). Furthermore, the intrinsic scarcity of MSCs exacerbates this challenge; for example, MSCs account for merely 0.001%–0.01% of total mononuclear cells in the bone marrow (188, 189). Therefore, extensive *in vitro* expansion is generally required to obtain clinically relevant cell numbers.

However, the *in vitro* expansion process introduces complications. MSCs expanded ex vivo may experience significant morphological changes, gene and protein expression profile modifications, and variations in their potential and physiological behaviors (190–192). Such changes can significantly affect the therapeutic use of MSCs and may even increase the risk of malignant transformation and tumor formation (18, 193). Indeed, while MSC-based therapies show significant promise for cancer treatment, evidence indicates that MSCs may contribute to tumor progression, local angiogenesis, metastasis, and drug resistance (194, 195).

A related issue is the poor engraftment and homing capacity of MSCs after administration. The delivery route significantly impacts therapeutic outcomes; for example, studies have shown that intravenous infusion can lead to the sequestration of MSCs in the capillaries of several organs, especially the lungs, liver, and spleen, which limits their effective distribution (16, 17, 196, 197). Furthermore, it is still poorly understood how MSCs behave and differentiate after being administered *in vivo*, with some studies suggesting that the surrounding microenvironment may trigger undesired differentiation or even pro-inflammatory behavior during the early inflammatory phase (19).

Nevertheless, inconsistencies in the isolation, expansion, and characterization of MSCs across laboratories continue to pose significant barriers. To tackle this issue, the Mesenchymal and Tissue Stem Cell Committee of the International Society for Cellular Therapy (ISCT) has established minimal criteria for defining MSCs, including plastic adherence, the expression of specific surface antigens, and the ability to differentiate into multiple cell lineages (14). Despite these guidelines, significant heterogeneity persists.

Published studies show that MSCs display greater variability in properties when subjected to plastic adherence and various culture media (198–200). Prolonged culture conditions further exacerbate this issue, often resulting in the loss of the MSCs' native supportive and anti-inflammatory functions (201). Genome-wide analyses have shown a dampening or downregulation of therapeutic gene expression profiles in cultured MSCs compared to their primary counterparts (202, 203). Additionally, *ex vivo*-expanded MSCs exhibit reduced expression of transcription factors crucial for producing paracrine factors, which diminishes their therapeutic potential. RNA sequencing data from human primary MSCs and their *in vitro* expanded counterparts have also indicated that primary MSCs maintain an enhanced hematopoietic supportive function (202).

Several strategies have been explored to address these challenges, including optimizing culture conditions by adjusting cytokine, glucose concentrations, and oxygen tension and employing three-dimensional culture systems such as mesenspheres (204). Alternatively, transcription factor-mediated reprogramming has been proposed for both murine and human MSCs to enhance their functional properties. This approach seeks to bolster the MSCs' anti-inflammatory response to *in vitro* stress and improve their hematopoietic supportive capacity (203, 205).

Ultimately, ethical and regulatory challenges persist, obstructing the clinical translation of MSC therapies. Issues regarding donor consent, cell ownership, manufacturing standards, and long-term safety monitoring must be addressed to guarantee responsible development and application (206–208).

## 5 Future direction: secretome and exosomes

Since MSCs primarily exert their effects through paracrine secretion, there has been a notable shift in MSC research toward the protective bioactive factors secreted by MSCs, such as the secretome, which has garnered considerable attention for its potential use in tissue repair and regeneration (209).

The secretome includes the factors and molecules secreted by stem cells into the extracellular space, such as soluble proteins, nucleic acids, lipids, and extracellular microvesicles. Based on their size and origin in the cell, these vesicles can be classified into three main categories: apoptotic bodies, microparticles, and exosomes (210).

The use of this cell-free therapeutic approach provides several advantages. Utilizing MSC-derived secretome as a lyophilized medical product could effectively alleviate concerns related to the infusion of *ex vivo* expanded cells as well as thawed and manipulated MSCs, presenting various biological and technological benefits. The secretome is favored over proliferating cells for safety reasons, including immune compatibility and tumorigenicity (210). The secretome derived from MSCs is also cost-effective because it eliminates the need for cell harvesting procedures (133).

A conditioned medium (CM) is a source of the secretome and vesicular elements used in regenerative therapies (210). The secretome's soluble components can be isolated through centrifugation, ion exchange chromatography, and filtration (211, 212). In the case of reprogrammed MSCs with enhanced paracrine activity, using the MSC secretome may alleviate any safety concerns associated with genetically modified cells.

The CM of MSCs is known to possess anti-inflammatory properties through various soluble molecules, such as TNF β1, IL-13, neurotrophin 3, ciliary neurotrophic factor, and IL-10 (213–215). Additionally, the CM also contains anti-apoptotic factors like bFGF and TGF, in addition to angiogenic factors, including VEGF and IGF-1 (21). Several studies have demonstrated the beneficial potential of CM in the context of various pathological conditions. For instance, CM derived from human cervical stem cells exhibited bactericidal activity against *S. epidermidis* and *E. coli* on infected corneal lenses (215). BM-MSCs-derived CM improved cortical neuron survival and neuronal connections *in vitro* and enhanced neurological recovery in a rat ischemia model. In particular, CM improved the survival of cortical neurons and facilitated the formation of neuronal connections in culture, while *in vivo* experiments revealed that the CM led to better neurological outcomes, suggesting its potential as a therapeutic option for neurological recovery (216). MSC secretome showed its efficiency in preventing and treating experimental NEC in mice and piglets by inhibiting TLR-4 signaling, reducing inflammation, and promoting intestinal remodeling and immune function, as shown by RNA sequencing (217). The TLR-4 pathway is well documented to contribute to the onset of pathology by facilitating the translocation of pathogenic bacteria, which subsequently elicits a pro-inflammatory response and leads to NEC development (218).

To proceed to administration in clinical trials, the secretome must be formulated into a standardized drug product that can be easily adopted by the clinical community (219). Several issues must be addressed, including the source, isolation methods, pharmaceutical quality controls, potency monitoring, toxicity, immunogenicity, administration route, and the definition of efficacy and long-term side effects (219, 220). Preclinical studies are necessary to identify the mechanisms of action of the components of the secretome. The diverse molecules carry various bioactive cargoes, including proteins, lipids, and metabolites, which can exert molecular and epigenetic effects on target cells. Additionally, the lack of standardized criteria for producing secretome presents a challenge. There is room for improvement in isolation methods to make more homogeneous preparations regarding particle number, potency, and purity (221).

Interest in the secretome for treating infant diseases like NEC has increased the focus on extracellular vesicles, especially exosomes (222). The term “exosome” refers to a distinct class of lipid membrane-bound extracellular vesicles, ranging in size from 40 to 150 nm in diameter and having a density between 1.09 and 1.18 g/ml (212). Many of the regenerative properties displayed by MSCs are mediated by secreted exosomes (223). Exosomes can deliver their contents to recipient cells through phagocytosis, fusion with the cell membrane, and receptor-ligand interactions (224). The lipid composition of these vesicles reflects their unique rigidity, and their function is to deliver bioactive lipids to recipient cells. Furthermore, exosomes contain genetic material, including messenger ribonucleic acid (mRNAs) and micro ribonucleic acid (miRNAs), which stimulate the degradation of their mRNA targets and circular ribonucleic acid (circRNAs) (225). Additionally, exosomes contain various cellular proteins, such as adhesion proteins, chaperones, and cytoskeletal proteins (226).

The recognition of exosomes as delivery vehicles for biological materials has prompted researchers to investigate their potential as therapeutic modalities, especially as drug carriers (227). Cell-derived vesicles provide several advantages over other drug delivery methods, including their natural composition, small size, and immune evasion capabilities that allow them to bypass the immune system (228). Extracellular vesicles can be loaded with exogenous cargo through several methods. The first method is electroporation, which uses an electric field from an electrode to create hydrophilic pores in the membrane, increasing membrane permeability and allowing substances with large molecular weights to pass through (229). Subsequently, transfection can be achieved by overexpressing a specific gene in the exosome donor cell or by treating a cell line with a drug of interest that will later be encapsulated in vesicles (230). The final technique is chemical-based exosome transfection, which uses commercially available transfection reagents to incorporate short interfering ribonucleic acid (siRNA) and deliver it to target cells via exosomes (231).

Extracellular vesicles from intestinal epithelial cells have been shown to activate wound repair mechanisms. Additionally, exosomes derived from these cells may affect antigen expression in the mucosal or systemic immune system through intercellular communication functions, thereby influencing NEC progression (167, 232, 233). Exosomes do not trigger human leukocyte antigen (HLA)-stem cell immune responses, making them less immunogenic than stem cells (234). The treatment of experimental NEC with exosomes derived from stem cells is as effective as treatment with stem cells themselves (111). Furthermore, exosomes from human umbilical cord mesenchymal stem cells reduced the severity of inflammatory bowel disease in mice by increasing IL-10 levels and decreasing TNF-α, IL-1β, and IL-6 in the colon tissues (109).

Research indicates that white matter injury observed in imaging studies contributes to adverse neurodevelopmental outcomes in children with NEC. Additionally, animal studies suggest that NEC-induced systemic inflammation may disrupt the blood-brain barrier (235, 236). In preclinical studies involving rodents, exosomes have been shown to traverse the blood-brain barrier; this has sparked interest in their therapeutic potential for NEC (237, 238). The paracrine effects of exosomes and their capacity to target brain injury present an intriguing therapeutic path for neuroprotection in NEC (239).

Rager et al. explored the protective role of exosomes derived from BM-MSCs against NEC. Administering them intraperitoneally to rat pups reduced NEC incidence and enhanced intestinal barrier function, showing no significant difference between the effects of exosomes and stem cells. This emphasizes that exosomes are likely the key mediators of the therapeutic effects of MSCs (240).

**Figure 1:**
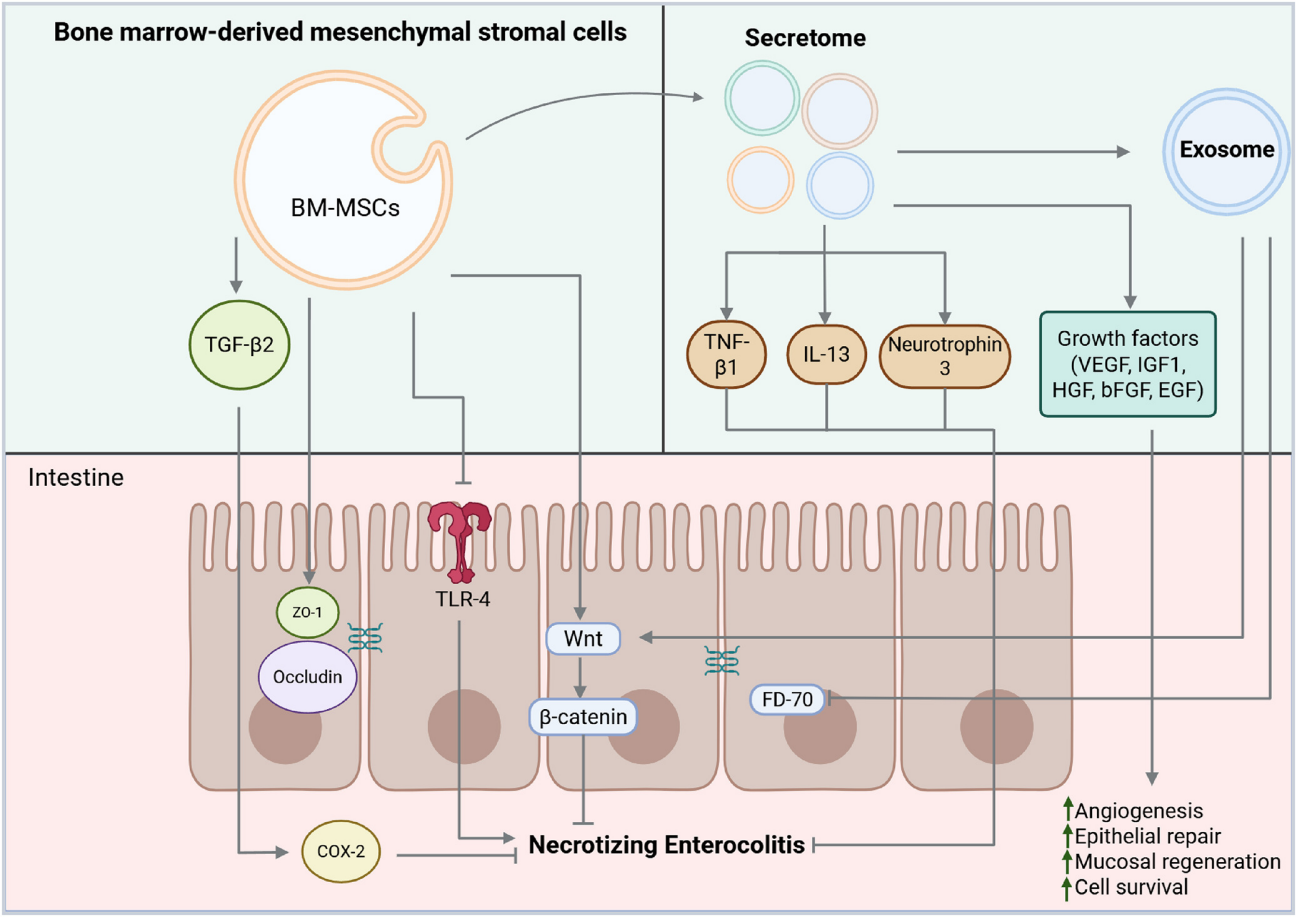
Functions and mechanisms of actions of bone marrow-derived mesenchymal stromal cells (BM-MSCs), secretomes, and exosomes in necrotizing enterocolitis (NEC). BM-MSCs act by activating transforming growth factor beta (TGF-β2), which, in a paracrine manner, triggers cyclooxygenase 2 (COX2), zonula occludens 1 (ZO-1) and occludin, thereby promoting the restoration of tight junctions. BM-MSCs impairs TLR4 pathway signaling thus interfering with NEC development. The secretome secretes a variety of bioactive molecules, including anti-inflammatory cytokines such as tumor necrosis factor β1 (TNF-β1), interleukin 13 (IL-13), and neurotrophin 3, which contribute to immune modulation. It also includes growth factors with potent regenerative and pro-angiogenetic properties, such as vascular endothelial growth factor (VEGF), insulin-like growth factor-1 (IGF-1), basic fibroblast growth factor (bFGF), hepatocyte growth factor (HGF), and epidermal growth factor (EGF). All these factors collectively enhance angiogenesis, epithelial repair, mucosal regeneration, and cell survival. Exosomes contribute to NEC protection by modulating the Wnt/β-catenin signaling pathway and improving intestinal permeability and gut barrier function by reducing the levels of FD-70 (70 kDa FITC-Dextran). Created with BioRender.com.

Figure 1 outlines the functions and mechanisms of action of NEC's BM-MSCs, secretomes, and exosomes.

## 6 Clinical translation prospects

Although MSCs have been investigated in several clinical trials, including those targeting rheumatic and autoimmune disorders, their large-scale clinical implementation remains complex (241, 242). In the context of necrotizing NEC, clinical translation is particularly challenging. A significant proportion of studies have explored the prophylactic use of MSCs in NEC, further amplifying the ethical concerns related to interventions in a highly vulnerable population such as preterm infants with multiple comorbidities. While cell-free strategies employing MSC-derived exosomes or secretomes offer a promising alternative by circumventing many of the inherent risks of cell-based therapies, their translational potential is currently limited by the lack of standardized protocols for their isolation, characterization, and quality control. Establishing universally accepted criteria for producing and validating these products is essential to reduce inter-study variability and ensure the safety and reproducibility required for future clinical application.

## 7 Conclusion

While the causes of NEC have been thoroughly studied, developing new therapeutic strategies has encountered several challenges because of the high incidence and mortality rates associated with this disease in preterm infants (98, 99). This highlights the ongoing need to examine additional aspects of NEC pathology. Due to their regenerative and anti-inflammatory properties, BM-MSCs are being reconsidered for treating neonatal diseases like NEC. In preclinical models, they secrete protective and reparative factors that reduce the incidence and severity of NEC. This mechanism includes the secretion of endocrine factors that aid tissue repair. While they enhance gut barrier function and lower intestinal permeability, whether these effects occur through shared or distinct molecular pathways remains unclear. Practical application addresses ethical, medical, and legal concerns (206).

Exosomes as therapeutic agents are promising candidates for developing novel strategies to treat NEC by reducing inflammation and intestinal permeability. Their ability to cross the blood-brain barrier and deliver therapeutic agents introduces a new concept for addressing NEC-related brain injury. Leveraging the therapeutic cargo and communication properties of exosomes enables us to explore cell-free therapy further while preserving the reparative potential of mesenchymal stromal cells (243). Despite their potential, the use of exosome-mediated therapy in NEC models remains limited. Therefore, further research is necessary to understand the safety, efficacy, and optimal administration methods for this treatment in NEC. Moreover, the type of stem cells, their origin, the various routes of administration, cell concentration, protocol duration, and starting point, combined with the marked variability in MSC isolation and culture methods, pose a substantial challenge for advancing this line of research in NEC. Standardization of these parameters is urgently needed to generate more robust and comparable results, ultimately facilitating their translation to the bedside. Enhancing our understanding of exosome biology in NEC could improve disease management and outcomes for premature infants globally.

## Glossary

53BP1: p53-binding protein 1
AF-NSCs: amniotic fluid-derived neural stem cells
AF-SCs: amniotic fluid-derived stem cells
ASCs: adult stem cells
bFGF: fibroblast growth factor
BM-MSCs: bone marrow-derived mesenchymal stromal cells
C34: 2-acetamidopyranoside
circRNAs: circular ribonucleic acids
CM: conditioned medium
DIC: disseminated intravascular coagulation
EGF: epidermal growth factor
ESCs: embryonic stem cells
ENS: enteric nervous system
FD70: 70 kDa FITC-Dextran
GA: gestational age
GIT: gastrointestinal tract
GvHD: graft-versus-host-disease
γH_2_AX: phosphorylated H2A histone family member X
HB-EGF: heparin-binding EGF-like growth factor
hESCs: human embryonic stem cells
HGF: hepatocyte growth factor
HLA: human leukocyte antigen
HMGB1: high mobility group box 1
HSPC: hematopoietic stem and progenitor cell
IBD: inflammatory bowel disease
IDO: indoleamine 2,3-dioxygenase
IGF-1: insulin-like growth factor
IL-1β: interleukin-1β
IL-6: interleukin-6
IL-10: interleukin-10
ISCT: international society for cellular therapy
LCA: lithocholic acid
MMC: migration motor complex
miRNAs: microribonucleic acids
mRNAs: messenger ribonucleic acids
MSCs: mesenchymal stromal cells
NCSs: neural stem cells
NEC: necrotizing enterocolitis
N-ENSCs: neonatal enteric neural stem cells
NF-kB: nuclear factor-kB
NICUs: neonatal intensive care units
NK: natural killer
Oct4: octamer-binding transcription factor 4
PGE: prostaglandin E2
P-MSCs: placental-derived mesenchymal stem cells
PXR: pregnane X receptor
siRNA: short interfering ribonucleic acid
SSEA-4: stage-specific embryonic antigen 4
TGF: transforming growth factor
Th1: T helper 1 cells
TLR-4: toll-like receptor 4
TPN: total parenteral nutrition
Treg: regulatory T cells
TUNEL: TdT-mediated dUTP nick-end labeling
UC-MSCs: umbilical cord-derived mesenchymal stem cells
VEGF: vascular endothelial growth factor
VLBW: very low birth weight
Wnt: wingless and Int-1
ZO-1: zonula occludens 1
